# Genetic Epidemiology, Hematological and Clinical Features of Hemoglobinopathies in Iran

**DOI:** 10.1155/2013/803487

**Published:** 2013-06-18

**Authors:** Zohreh Rahimi

**Affiliations:** ^1^Medical Biology Research Center, Kermanshah University of Medical Sciences, Daneshgah Avenue, P.O. Box 67148-69914, Kermanshah, Iran; ^2^Department of Biochemistry, Medical School, Kermanshah University of Medical Sciences, Daneshgah Avenue, P.O. Box 67148-69914, Kermanshah, Iran

## Abstract

There is large variation in the molecular genetics and clinical features of hemoglobinopathies in Iran. Studying structural variants of hemoglobin demonstrated that the *β*-chain variants of hemoglobin S and D-Punjab are more prevalent in the Fars (southwestern Iran) and Kermanshah (western Iran) provinces, respectively. Also, **α**-chain variants of Hb Q-Iran and Hb Setif are prevalent in western Iran. The molecular basis and clinical severity of thalassemias are extremely heterogenous among Iranians due to the presence of multiethnic groups in the country. *β*-Thalassemia is more prevalent in northern and southern Iran. Among 52 different *β*-thalassemia mutations that have been identified among Iranian populations, IVSII-1 G:A is the most frequent mutation in most parts of the country. The presence of IVS I-5 G:C mutation with high frequency in southeastern Iran might reflect gene flow from neighboring countries. A wide spectrum of **α**-thalassemia alleles has been detected among Iranians with −*α* ^3.7 kb^ as the most prevalent **α**-thalassemia mutation. The prevention program of thalassemia birth in Iran has reduced the birth rate of homozygous *β*-thalassemia since the implementation of the program in 1997. In this review genetic epidemiology, clinical and hematological aspects of hemoglobinopathies, and the prevention programs of *β*-thalassemia in Iran will be discussed.

## 1. Introduction

Inherited disorders of hemoglobin, hemoglobinopathies, are the most common monogenic diseases. They are a group of autosomal recessive disorders that are classified to two main groups of thalassemia syndromes (*α*- and *β*-thalassemia) and structural variants of hemoglobins [1]. The presence of a structural variant along with reduced synthesis of hemoglobin is observed in Hb E [2]. The wide variation in the clinical manifestation of hemoglobin disorders could be attributed to the influence of various genetic modifiers and environmental factors. Heterogenous distribution of the disease and the presence of high variation in the phenotypic manifestation of a specific mutation are major problems with the development of programs for the control of the hemoglobinopathies [1]. High frequency of the hemoglobinopathies in some populations, especially in many tropical countries, reflects heterozygote advantage against severe malaria [1]. Also, consanguineous marriage plays an important role in high frequency of these disorders [3]. Iran a country with medium malaria endemicity and high rates of consanguineous marriage consisted of different ethnic groups with various prevalence and clinical feature of hemoglobinopathies. So, the knowledge of genetic epidemiology of hemoglobinopathies in the country will be of valuable help in the development of successful prevention programs and better diagnosis and management of hemoglobin disorders in Iran. 

## 2. Malaria and Hemoglobinopathies

Malaria disease is transmitted by anopheline mosquitoes. This disease is highly distributed in tropical and subtropical regions. Malaria hypothesis suggested by Haldane was based on the high carrier frequency of hemoglobinopathies in areas with malaria endemicity; a phenomenon protects carriers against malaria and might be due to the balanced polymorphism in human populations [4]. Epidemiologic, in vitro, and in vivo studies support association between structural variants of Hb S, Hb C, and Hb E as well as *α*- and *β*-thalassemia with areas of prevalence of *Plasmodium falciparum* [5].

The Hb S variant decreases the risk of infection by *P*. *falciparum*. The mechanism of red blood cells (RBC) resistance in sickle cell trait (AS) individuals could be due to the lack of parasite development and accelerating Hb S polymerization in low O_2_ tension and higher rates of phagocytosis of parasite-infected sickle erythrocyte by host immune cells [4, 5]. The Hb AS protects against all forms of malaria by elimination of parasites in 90% of Hb S cell populations. In Hb S carriers the selection occurs at the cost of the loss of homozygotes from sickle cell disease [4, 6].

The selective advantage of Hb C is greater in homozygous state (90%) than in Hb AC carriers (30%) [6].

The protection of *β*-thalassemia patients against malaria is due to delay of switching from fetal to adult hemoglobin resulting in higher level of intracellular Hb F. The Hb F is more stable and stronger than Hb A and is partially resistant to digestion by proteolytic machine of parasite. So, Hb F containing red cells are inadequate hosts for *P*. *falciparum,* and this hemoglobin protects the host from the lethality of malaria [5]. Also, the presence of higher levels of serum antibodies bound to *β*-thalassemic erythrocytes is the other mechanism provides the protection against malaria in these patients [4].

*α*-Thalassemia is very prevalent in malaria endemic regions which protects against severe forms of *P*. *falciparum* infection. The infected erythrocytes of *α*-thalassemia patients have high levels of antibodies on their surface that promote antiparasite effects [4]. The protection of *α* ^+^-thalassemia against malaria might be due to higher susceptibility of homozygotes to malaria in early life that immunize these individuals and make them resistance to malaria later [7]. The protective effect of *α*-thalassemia is specific to severe malaria [6].

Iran as an eastern Mediterranean country with medium malaria endemicity [8] started the program of malaria eradication in 1956. Later in 1980 the program changed to malaria control program. Now this disease is mostly restricted to three southeastern provinces of Sistan-Baluchestan, Hormozgan, and Kerman [9]. According to WHO malaria report Iran is classified in the preelimination stage of malaria [10]. Due to heterozygote advantage against *P*. *falciparum* malaria there is a high incidence of the *β*-thalassemia in Iran especially in northern and southern Iran as the ten provinces with high prevalence of *β*-thalassaemia are Gilan, Hormozgan, Khuzestan, Kohgiluyeh-Boyer-Ahmad, Fars, Bushehr, Sistan-Baluchestan, Kerman, and Isfahan in northern and southern Iran [11, 12]. The Sistan-Balouchestan province that is the most prevalent area for malaria comprises 42–60% of total malaria cases of Iran. Majority of infected cases (85%) with malaria were due to *P*. *falciparum* [13]. Isfahan province located in central Iran has been classified as the sixth more prevalent province for malaria after Sistan-Balouchestan, Hormozgan, Kerman, Fars, and Tehran provinces [14].

The gene frequency of *β*-thalassaemia has been estimated to be 0.0174 with over 2.25 million carriers [15]. An overall rate of consanguineous marriage (majority first-cousin marriages) is 38.6% (ranges from 16 to 47%) in Iran [15]. 

## 3. Structural Variants

### 3.1. Hemoglobin S

The hemoglobin (Hb) S that results from glutamic acid→valine amino acid substitution at position 6 of *β*-chain is the most clinically important structural variant of hemoglobin with highest frequency in Africa, Saudi Arabia, and India. Sickle cell disease (SCD) results from the homozygous state of the mutation, or a compound heterozygous state with one of structural variants of Hb D-Punjab, Hb O-Arab, Hb C, or *β*-thalassemia mutation in the other *β*-genes [16, 17].

First report of clinical and hematological features of sickle cell anemia in southern Iran demonstrated the high level of Hb F (18%) and mild clinical course of the disease in Iranian patients [18]. The prevalence of sickle cell trait and sickle cell anemia in southern Iran has been estimated to be around 1.43 and 0.1%, respectively [19]. In central Iran (southeast Isfahan) sickle cell trait was reported with a frequency of 8.33% [20]. In contrast, in western Iran the lowest frequency for this hemoglobin variant compared to structural variants of Hb D-Punjab, Hb Q-Iran, and Hb Setif was reported [21].

### 3.2. Hb D-Punjab

Hb D-Punjab (D-Los Angeles) is characterized by substitution of glutamine for glutamic acid at position of 121 of *β*-globin chain [22]. The presence of Hb D-Punjab in combination with an *α*-chain variant (Hb O-Indonesia) in an Iranian patient was reported 37 years ago by Rahbar et al. [23]. The case had no hemolytic disorders and clinical symptoms. Subsequent report indicated that in Kurdish population from western Iran Hb D-Punjab was the most prevalent structural *β*-globin variant [22]. 

Hb D-Punjab was detected as the second prevalent structural variant in southern province of Khuzestan [24]. Mild clinical presentation of this variant in homozygous and combined heterozygous state with a *β* ^0^-thalassemic mutation and also with concomitant presence of *α*- and *β* ^0^-thalassemia mutations have been indicated [22]. Benign feature of Hb D-Punjab was confirmed by two separate reports of concomitant presence of this structural variant with *β*-thalassemia mutation of IVSII-1 G:A that produced a minor *β*-thalassemia picture [25, 26].

### 3.3. Hb Q-Iran

Single nucleotide mutations in *α*1 or *α*2 genes produce abnormal *α*-chain hemoglobins. Hb Q disorders including Hb Q-Iran [*α*75 (EF4) Asp→His], Hb Q-Thailand [*α*74 (EF3) Asp→His], and Hb Q-India [*α*64 (E13) Asp→His] are important Hb variants. All of these hemoglobins are slow moving variants that migrate at the electrophoretic position of Hb S at alkaline pH [27, 28]. In carriers of Hb Q-Iran this hemoglobin has a level of 17–19% and hematological indices are normal [29]. In recent studies from Kurdish population of western Iran this Hb variant was the second prevalent structural variant with a level of 14.2–29.3% and in carriers of this hemoglobin hematological indices were normal [21, 29]. 

### 3.4. Hb Setif

Hb Setif [*α*94 (G1) Asp→Tyr] is an *α*-chain Hb variant with electrophoretic mobility similar to Hb S at alkaline pH. Hb Setif is much less soluble than Hb A and induces pseudosickling of the red cells in vitro. This hemoglobin presents in a low percent (12–17%). Hb Setif has been found in Algeria, Iran, Lebanon, Saudi Arabia, Turkey, Italy, Malta, and Cyprus. This *α*-chain variant was found to be third prevalent structural variant among Kurdish population from western Iran with a level of 10.8–27.1% and normal hematological indices in carriers of the hemoglobin [29].

### 3.5. Epistatic Modifiers and Structural Variants

#### 3.5.1. Hb S

The *β* ^S^ mutation has been found to be in linkage disequilibrium with five distinct typical (common) *β*-globin gene cluster haplotypes. Four of these haplotypes are known as African haplotypes (Bantu, Benin, Senegal, and Cameroon), the fifth is the Arab-Indian haplotype, which was originally described in the eastern oases of Saudi Arabia and among the tribal Adivasi population of India [30]. In Iran genetic studies and determination of haplotype background of *β* ^S^ gene, for the first time, were conducted in central Iran which indicated that the *β* ^S^ gene was in linkage disequilibrium with the Arab-Indian haplotype in this area [20]. Subsequently, in southwestern Iran the association of *β* ^S^ gene with Arab-Indian haplotype was reported [31]. Mild clinical presentation of sickle cell anemia in southwestern Iran has been demonstrated with high level of Hb F and the elevation ratio of ^G^ *γ* : ^A^ *γ* chains in sickle cell anemia patients that was attributed to the presence of Xmn I polymorphic site associated with Arab-Indian haplotype [31, 32]. More analysis of *β* ^S^ gene from different provinces of southwestern Iran demonstrated that the most prevalent haplotype in various ethnic groups (Fars and Arabs) was the Arab-Indian haplotype [30]. However, around 33% of *β* ^S^ genes were correlated with African haplotype. These findings provided an evidence of multicentric origin of *β* ^S^ gene in southwestern Iran. The prevalence of −*α* ^3.7 kb^ mutation among SCD patients from southwestern Iran was around 37%. The beneficial effect of the presence of *α* ^+^-thalassemia in SCD patients of southwestern Iran was appeared in lower levels of MCH and MCV [32]. In western Iran the *β* ^S^ gene was in linkage equilibrium with Benin haplotype [21].

#### 3.5.2. Hb D-Punjab

Molecular genetic studies indicated an association between *β* ^D^-Punjab gene with haplotype I and its unicentric origin in western Iran [22]. However, in two southern provinces of Fars and Hormozgan four haplotype backgrounds for *β* ^D^-Punjab gene with the most prevalent haplotype I (67.5%) have been observed [33].

#### 3.5.3. Hb Q-Iran

Higher expression of Hb Q-Iran with lower levels of MCV in carriers of this hemoglobin was observed with concomitant presence of this variant with −*α* ^3.7 kb^ mutation [21]. It has been suggested that Hb Q-Iran is a benign structural variant of Hb, which in combination with a *β* ^0^-thalassemia mutation and in the presence of *α* ^+^-thalassemia, produces only a minor *β*-thalassemia picture with moderate anemia and elevation of Hb F [34].

## 4. Thalassemia

Thalassaemias are characterized by decrease or absence production of *α*- or *β*-globin chains, which result in two main types of *α*- or *β*-thalassemia [35]. More than 200 different types of deletional and nondeletional mutations in *α*- or *β*-globin genes have been detected with diverse clinical manifestations, ranging from asymptomatic to profound fatal anemias in utero [36].

### 4.1. *β*-Thalassemia

*β*-Thalassemia has a high prevalence (around 10%) in north, close to the Caspian sea and South of Iran close to the Persian Gulf. The prevalence of *β*-thalassemia alleles in most parts of the country has been estimated to be 4–8% [37, 38]. Overall, there are 20,000 homozygote and 3,750,000 carriers of *β*-thalassaemia in Iran [39].

Finding 52 different mutations among Iranians and the presence of different mutations in 21% of consanguineous couples suggest the heterogeneous picture of *β*-thalassaemia mutations among Iranian populations [15]. So, many studies in various parts of Iran have been performed to establish the spectrum of *β*-thalassemia mutations to help the prenatal diagnosis.

#### 4.1.1. Southern Iran

In reports from three ethnic groups of Southern provinces of Fars, Hormozgan, and Khuzestan different spectrum and prevalence of *β*-thalassemia mutations were obtained [24, 37, 40–42]. In the Fars province (with ethnic background of Fars) IVS II-1 G:A and IVS I-6 T:C were the most prevalent *β*-thalassemic mutations with the frequencies of 31 and 15%, respectively, among 10 different mutations that were found in 26 studied chromosomes that suggested a genetic admixture and migrations for the presence of high number of *β*-thalassemic mutations in this area [40]. Second and third reports from this area confirmed the presence of IVS II-1 G:A as the most prevalent *β*-thalassemic mutation that was associated with Xmn I polymorphic site [37, 41]. However, in other southern province of Hormozgan (with various ethnic groups of Fars, Arab, and Balouch) neighboring the Fars province the IVS I-5 G:C with a frequency of 69% was the most prevalent mutation followed by the IVS II-1 G:A (9.6%). In contrast with other provinces of Iran no IVS I-110 G:A or IVS I-1 G:A mutations were found in this area. Also, the severe phenotype of patients homozygous for the IVS II-745 C:G mutation was linked in cis to the 5′UTR +20 C:T transition [42]. In more recent study from Hormozgan province among 224 *β*-thalassaemia carriers mostly with Fars origin (98%), the IVS I-5 G:C mutation with a frequency of 71% was the most prevalent mutation followed by IVS II-1 G:A (12%) [12]. In this province due to malaria selection and high rates of consanguineous marriages hemoglobinopathies are common [12]. In another southern province of Khuzestan with Arab ethnic background three most common mutations including CD 36/37-T, IVS-II-1 G:A, and IVS-I-110 G:A at a frequency of 20.5, 20.0, and 14.2%, respectively, were detected [24]. The picture of *β*-thalassaemia mutations in this province is heterogenic with detecting 42 different types of mutations [24].

#### 4.1.2. Northern Iran

In three northern provinces of Mazandaran, Gilan, and Golestan, IVS-II-1 G : A with a frequency of 50% was the highest prevalent mutation followed by CD 30 G:C (7.7%). The CD 8 −AA, CD 22/23/24 –AAGTTGG, and IVS-I-5 G:C were the other common mutations in the area [43]. Similarly, in other studies from these northern provinces the IVS-II-1 G:A was the most prevalent mutation (56.1%) and the CD 30 G:C (8.1%) was the second prevalent mutation. However, CD 8/9 +G (6.1%), CD 22/23/24 –AAGTTGG (3%), IVS-I-110 G:A (2.5%), IVS-I-5 G:C (2.3%), and IVS-II-745 C:G (2.3%) were the other common mutations [44]. High prevalence of IVS-II-1 G:A in northern Iran compared to other parts of Iran might be attributed to high rates of consanguineous marriages in this area [44]. In subsequent study from Mazandaran the frequency of IVS-II-1 G:A mutation as the most prevalent mutation was 61% [45], and the CD 30 G:C with a frequency of 7.5% was the second prevalent mutation. Also, in provinces of East Azerbaijan and Ardabil, northwestern, the most frequent mutations in order of frequencies were IVS-II-1 G:A (21%), IVS-I-110 G:A (18%), CD 8/9 +G (14.5%), CD 8 −AA (8%), and IVS-I-1 G:A (7.5%) [46].

In northeastern province of Khorasan in a small subset of chromosomes the CD 8/9 +G was the most frequent mutation (62.5%), and each of the three mutations of IVS-II-1 G:A, 36/37 –T, and CD 39 C:T had a frequency of 12.5% [43]. 

#### 4.1.3. Western Iran

The spectrum of *β*-thalassemia mutations in western Iran provinces of Kermanshah (mostly Kurds), Kurdistan (with Kurdish ethnic background), Lorestan (mostly Lors), Ilam (mostly Kurds), and Hamadan (mostly Fars) were identified [43, 47–49]. In the Kermanshah province twenty different mutations were identified that among them around 81% were of *β* ^0^ type mutation. Four most common mutations in this part of Iran were IVSII-1 G:A (32.97%), CD8/9 +G (13.51%), IVSI-110 (G:A) (8.38%), and CD 36/37 −T (7.84%) [47]. Test of heterogeneity between different Iranian populations indicated that Kermanshah population is significantly different from the population of southern Iran (*P* < 0.004), degree of freedom (df = 7), but considering the most frequent mutations, the spectrum of *β*-thalassemia mutations in Kermanshah is similar to people from Lorestan, Ardabil, East Azerbaijan, Gilan, Mazandaran, Golestan, Tehran, and Isfahan [47].

The spectrum and frequency of *β*-thalassemia mutations in Kurdistan province have been found to be similar to the Kermanshah province. In the Kurdistan province IVS-II-1 G:A was the most frequent, comprising 35% of all mutations. Other common mutations were CD 8/9 +G (15.7%), IVS-I-1 G:A (8%), CD 5 −CT (6.7%), CD 8 −AA (6.7%), and IVS-I-110 G:A (6%) [48]. In the Lorestan province CD 36/37 −T mutation, with a frequency of 33.8%, was the most common mutation. This mutation has been detected among Kurdish Jews [43]. The other most frequent mutations were IVS-II-1 G:A, IVS-I-110 G:A, and CD 8/9 +G with the frequencies of 27.7, 11.5, and 10.8%, respectively [49]. In two other western provinces of Hamadan and Ilam IVSII-1 G:A (29.4%) was the most prevalent mutation, and IVSI-110 G:A and CD 8 −AA were the second and third prevalent mutations with frequencies of 19.6 and 11.7% [43].

#### 4.1.4. Central Iran

In a study from central provinces of Tehran, Isfahan, Yazd, Markazi, and Semnan the three most common mutations were IVS-II-1 G:A (28%), CD 8/9 +G (11.7%), and IVS-I-5 G:C (9.3%) [43]. In a subsequent report from the Isfahan province the presence of IVS-II-1 G:A (20.5%) as the most prevalent mutation was confirmed. However, the second prevalent mutation was IVS-I-5 G:C (11%) [50]. 

#### 4.1.5. Southeastern Iran

The high frequency of *β*-thalassemia alleles in the Sistan-Balouchestan province could be attributed to the selection pressure by malaria and high rates of consanguineous marriage among Balouch population. Around 90% of homozygous *β*-thalassemia patients of the province have ethnic background of Balouch [51]. 

In this province among Balouch population only IVS I-5 G : C with an unusual frequency of 87.2% along with CD 8/9 +G with a frequency of 4% constituted about 91% of *β*-thalassemia mutations. The IVS I-5 G:C has been found as the most prevalent mutation among Sindh and Balouchestan provinces of Pakistan bordering Sistan-Balouchestan province of Iran [51]. Also, in southeastern province of Kerman IVS I-5 G:C was detected to be the high prevalent *β*-thalassemic mutation (66.2%) followed in order of frequency by IVS II-1 G:A (6%) and CD 8/9 +G (4.9%) [52].

The spectrum of *β*-thalassemia mutations in various parts of Iran indicating the IVSII-1 G:A as the prevalent mutation in most part of Iran with the highest frequency in northern Iran and decreased its frequency toward southern Iran where IVS I-5 G:C is the highest prevalent mutation. The high frequency of Mediterranean mutation IVSII-1 G:A in Iran might be due to an independent origin of this mutation or genetic admixture. The Balouchestan province of Pakistan is known for the highest frequency of IVSI-5 G:C. The presence of this mutation as the most prevalent mutation in Southeastern Iranian province of Sistan-Balouchestan might be due to genetic admixture with the population of neighbor country of Pakistan. The same reason could explain the similar frequency of IVS I-5 G:C (69%) between southern Iranian province of Hormozgan and the southern neighbor of United Arab Emirates (66%) [43].

#### 4.1.6. Epistatic Modifiers and *β*-Thalassemia

Genetic variants that results in differences in disease phenotype are known as modifier genes [53]. These modifiers include the presence of *α*-thalassemia (with reducing the globin chain imbalance) and/or the presence of genetic determinants (with increasing the production of Hb F) in adult life [53]. High frequency of *α*-thalassemia is observed in many populations with high prevalence of *β*-thalassemia [54]. The presence of *α*-thalassemia in homozygous or compound heterozygous state of *β*-thalassemia ameliorates the clinical presentation of the disease due to the lower concentration of excess *α*-globin chains [55].

The IVS II-1 G:A among Kurdish population of Kermanshah province (western Iran) was strongly associated with haplotype III [56]. However, this mutation in southern Iran was mostly associated with haplotype I [57]. A frequency of 0.39 was obtained for the Xmn I polymorphic site in *β*-thalassemia major patients of western Iran associated with elevation in ^G^ *γ*-chain level and ^G^ *γ* : ^A^ *γ* ratio and improved clinical features [58]. The increased numbers of *γ*-chains bind to excess *α*-globin chains produce Hb F and reduce globin chain imbalance [55]. Further, the main molecular basis of the *β*-thalassemia intermedia phenotype in Iranian cases was determined to be the coinheritance of a positive Xmn I polymorphism with *β*-globin mutations resulted in increase Hb F production, coinheritance of *α*-globin defects, and mild presentation of *β*-globin mutations [59]. Also, HS-111 and 3′HS1 in the promoter region of *γ*-globin gene in *β*-thalassemia intermedia patients were associated with high level of Hb F [60].

### 4.2. *α*-Thalassemia

The *α*-thalassemias are classified into the *α* ^0^-thalassemia with no synthesis of *α*-chains and *α* ^+^-thalassemia with a reduction in synthesis of *α*-chains. The heterozygous and homozygous *α* ^0^-thalassemia are designated as −/*αα* and −/−, respectively. *α* ^+^-Thalassemia in heterozygous and homozygous states is represented as −*α*/*αα* and −*α*/−*α*, respectively [61]. The severe *α*-thalassaemia mutations are restricted to Southeast Asia and some of the Mediterranean islands. The *α* ^+^-thalassemia deletions are the most common forms of *α*-thalassemia with two major forms of −*α* ^3.7 kb^ and −*α* ^4.2 kb^ [1, 61]. Also, more than 70 nondeletional forms of *α*-thalassemia have been detected that might be coinherited with deletional mutations or other genetic modifiers results in diverse genotypic and/or phenotypic expressions [36].

Identification of molecular spectrum of *α*-thalassemia in southern Iran (Hormozgan province) revealed the two common alleles of −*α* ^3.7 kb^ (79.1%) and *α* ^−5nt(−TGAGG)^ (4.3%) in this area [62]. Also, the presence of −*α* ^3.7 kb^ single gene deletion as the most frequently *α*-chain variant in southwestern provinces of Fars, Khuzestan, and Kohgiluyeh-Boyer-Ahmad was confirmed [63, 64].

In northern provinces of Mazandaran and Gilan 21 and 16 different *α*-globin alleles, respectively, were found that among them −*α* ^3.7 kb^ with frequencies of 44.9 and 42.5%, respectively, and *α* ^PolyA2(AATGAA)^ with frequencies of 18.2 and 12.4%, respectively, were the most prevalent mutations in this area. The frequency of *α* ^−5nt(−TGAGG)^ was 6.5% in Mazandaran and 7.1% in Gilan provinces [65, 66].

In Kerman province from southeastern Iran the spectrum of *α*-thalassemia mutations indicated −*α* ^3.7 kb^ as the most common mutation (83.8%) followed by *α* ^CD19(−G)^ (5.7%) and *α* ^−5nt(−TGAGG)^ (4.2%) [67]. In some cities of Kerman province such as Jiroft and Kahnooj with malaria endemicity the highest frequencies of *α*-thalassemia has been detected [67].

Overall, studying 8 different geographic areas of Iran indicated the presence of 16 different *α*-globin mutations that among them the −*α* ^3.7 kb^ mutation was found to be the most frequent *α*-thalassemia mutation, comprising 60.2% of *α*-thalassemia alleles. The highest frequency of this mutation was observed in south (93%), and the lowest frequency of the mutation was detected in central (53.9%) Iran. Nine of these mutations including −MED, −*α* ^4.2 kb^, *α* ^PolyA2(AATGAA)^, *α* ^CS^, *α* ^−5nt(−TGAGG)^, −*α* ^20.5^, *α* ^PolyA1(AATAAG)^, *α* ^CD19^, and *α* ^CD59^ reached to a polymorphic frequency (>1%) [68].

Hb H (−/−*α*) is an unstable Hb resulting from deletion of three *α*-globin genes and has a mild-to-moderate severe phenotype [69]. This Hb forms intracellular precipitates that results in early cell death. Hemolysis rather than ineffective erythropoiesis is the primary cause of anemia in Hb H disease. Many patients require intermittent transfusions. The clinical severity is strongly influenced by the type of mutation. Among Iranians the clinical diversity of Hb H disease (similar genotypes with different phenotypes) has been reported [70]. Deletions on chromosome 16 are responsible for 75% of Hb H mutations, and these deletions cause a milder form of the disorder. The remaining 25% of patients with Hb H disease have two deletions plus a point mutation or insertion in the *α*-globin gene. Nondeletional Hb H is often severe and likely requires transfusions. Hemoglobin Constant Spring (Hb CS) is the most common nondeletional *α*-thalassemia mutation associated with Hb H disease. The gene frequency of Hb CS reaches to 8% in Southeast Asia. The laboratory and clinical course of Hb H/CS disease are more severe than Hb H disease [71].

Studying molecular basis of Hb H disease in southwest Iran indicated the presence of six genotypes associated with Hb H disease in the area including −*α* ^3.7 kb^/−MED, −*α* ^3.7 kb^/*α* ^T-Saudi^ *α*, *α* ^T-Saudi^ *α*/*α* ^T-Saudi^ *α*, *α* ^CS^ *α*/−MED, −MED/*α* ^T-Turkish^ *α*, and the atypical forms of Hb H disease −*α* ^3.7 kb^/*α* ^CS^ *α*. It has been suggested that the molecular background of Hb H disease in the southwest area of Iran is more similar to the Mediterranean type than to the Southeast Asian [72]. 

A study conducted on patients with Hb H disease to find the association between severity of phenotype according to Hb H genotype. This study revealed a subset of Hb H genotypes, including −MED/*α* ^CS^ *α*, −MED/*α* ^PolyA2(AATGAA)^ *α*, and *α* ^CS^ *α*/*α* ^CS^ *α* that was associated with a need for regular or irregular blood transfusions [73].

**Figure 1:**
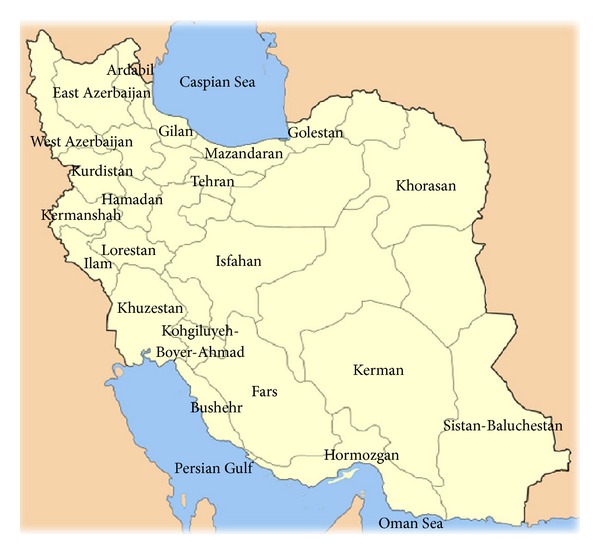
Map of Iran depicts provinces with prevalence of hemoglobinopathies.

A map of Iran that depicts provinces with prevalence of hemoglobinopathies is presented in Figure 1. Also, the common types of hemoglobinopathies in different areas of Iran are demonstrated in Table 1.

## 5. Prevention Programs

Due to large economic burden of treatment of *β*-thalassaemia patients for both affected individuals and national health budget a mandatory premarital screening program for *β*-thalassaemia was established [15]. Preventing programs of *β*-thalassaemia in Iran are more cost-effective than treatment of the disease [11].

In Iran, public education, screening, and prenatal diagnosis remarkably decreased the frequency of births of babies with severe forms of *β*-thalassaemia. Thalassemia carrier screening started from 1991 in Shiraz and then from 1995 in all provinces of Iran with the aims to reduce the burden of disorder by identifying those individuals at increased risk and to determine their reproductive risks and having an affected child. In 1997, the technical committee of prenatal diagnosis (PND) of *β*-thalassemia in the Ministry of Health and Medical Education was established to define the standard protocols for PND that resulted in a reduced rate of *β*-thalassemia incidence in the country [74]. At first the only method for prevention of *β*-thalassemia was premarital screening. In 2001, the laws of the country were modified to permit abortion of affected fetuses [15] then from 2002 the abortion before 16-week gestational age was possible [39]. In southern and northern provinces of Iran where the prevalence of *β*-thalassemia is high the results of this program have been successful in decreasing the birth prevalence of *β*-thalassemia. However, the incidence of this condition has not been eliminated yet [39]. In this program, it is required that every couple to be tested for *β*-thalassemia by their red blood cell indices in order to receive a permit for marriage registration. If the results of the previous test were conspicuous, the levels of hemoglobin A_2_ and Hb electrophoresis were also determined. Couples who were both carriers received counseling. For those who ignored the recommendation and decided to marry, prenatal diagnosis and termination of pregnancy in case of an affected fetus have been offered [35, 74]. During this period the average of high risk couple initially deciding not to marry was 90% without detection of new cases of thalassemia [75]. Also, the trend for prenatal diagnosis among at-risk couples of Sistan-Balouchestan significantly increased from 2002 to 2010 [51]. Thalassemia screening program in Iran is a well-coordinated national approach in which screening is integrated with services for patients [76].

## 6. Conclusion

The molecular basis and clinical severity of *α*- and *β*-thalassemia are extremely heterogenous among Iranians due to the presence of multiethnic groups in the country. The presence of Mediterranean *β*-thalassemic mutation of IVSII-1 G:A with highest frequency in most parts of the country might be attributed to the independent origin of this mutation or genetic admixture. However, the presence of IVS I-5 G:C mutation with high frequency in southeastern Iran might reflect gene flow from neighboring countries. A wide spectrum of *α*-thalassemia alleles has been detected among Iranians with −*α* ^3.7 kb^ as the most prevalent *α*-thalassemia mutation. There are few available studies on the frequency, genetic and clinical features of structural variants of Hb in Iran. All reports are from southwestern and western Iran indicated that the Hb S and Hb D-Punjab, respectively, have been studied in details as the most prevalent structural variants in these parts of the country. The prevention program of thalassemia birth in Iran by premarital screening, prenatal diagnosis, and termination of pregnancies has been a successful policy in reducing birth rate of homozygous *β*-thalassemia and a large amount of medical expenses in Iran since the implementation of the program in 1997. However, it is still more work to achieve zero *β*-thalassemic birth.

## Figures and Tables

**Table 1:** Common types of hemoglobinopathies in various parts of Iran.

Area	*α*-Chain variants	*β*-Chain variants	*α*-Thalassemia	*β*-Thalassemia
Northern Iran				
Gilan			−*α* ^3.7 kb^ (42.5%)	IVS II-1 G : A (52.4%)
Mazandaran			−*α* ^3.7 kb^ (44.9%)	IVS II-1 G : A (62.1%)
Golestan				IVS II-1 G : A (33.3%)
Northwestern Iran				
East Azerbaijan and Ardabil				IVS II-1 G : A (21%)
West Azerbaijan				IVS II-1 G : A (35%)
Northeastern Iran				
Khorasan				CD 8/9 +G (62.5%)
Central Iran				
Isfahan		Hb S		IVS II-1 G : A (20.5 and 28%)
Southwestern Iran				
Fars		Hb S	*α* ^ 3.7 kb^ (71.7%)	IVS II-1 G : A (24, 31 and 47.8%)
Khuzestan		Hb S Hb D-Punjab	−*α* ^3.7 kb^ (62.6%)	CD 36/37 –T (20.5%) IVS II-1 G : A (20%)
Western Iran				
Kermanshah	Hb Q-Iran Hb Setif	Hb D-Punjab		IVS II-1 G : A (33%)
Kurdistan				IVS II-1 G : A (35%)
Lorestan				CD 36/37 –T (33.8%) IVS II-1 G : A (27.7%)
Southern Iran				
Hormozgan			−*α* ^3.7 kb^ (79.1%)	IVS I-5 G : C (69%)
Southeastern Iran				
Sistan-Balouchestan				IVS I-5 G : C (87.2%)
Kerman			−*α* ^3.7 kb^ (83.8%)	IVS I-5 G : C (66.2%)
Iranians (with different ethnicity)			−*α* ^3.7 kb^ (60.2%)	IVS II-1 G : A (34%)

## Genetic Terms

Hb D-Punjab (D-Los Angeles): A structural variant of *β*-globin chain (CD 121 Glu→Gln)
Hb E: A structural variant of *β*-globin chain (CD 26 Glu→Lys) results in reduced synthesis of *β*-globin chain
Hb Q-Iran: A structural variant of *α*-globin chain (*α*75 (EF4) Asp→His)
Hb Setif: A structural variant of *α*-globin chain (*α*94 (G1) Asp→Tyr)
Hb S: A structural variant of *β*-globin chain (CD 6 Glu→Val)
Hb H: Deletion of three *α*-globin genes (−/−*α*)
*α*^+^-Thalassemia: Reduced synthesis of *α*-globin chains
*α*^0^-Thalassemia: Complete absence of synthesis of *α*-globin chains
*β*^+^-Thalassemia: Reduced synthesis of *β*-globin chains
*β*^0^-Thalassemia: Complete absence of synthesis of *β*-globin chains.
